# Diversity and Symbiotic Associations of Endophytic Fungi in *Calotropis procera* (Aiton) W.T. Aiton (Asclepiadaceae) Across Three Egyptian Regions: Phenotypic Characterization and Mitotic Activity

**DOI:** 10.1007/s00248-025-02503-6

**Published:** 2025-03-05

**Authors:** Hoida Zaki, Mohamed A. Hussein, Eman G. A. M. El‑Dawy

**Affiliations:** 1 Botany and Microbiology Department, Faculty of Science, South Valley University, Qena, Egypt; 2 Applied and Environmental Microbiology Center, South Valley University, Qena, Egypt

**Keywords:** *Calotropis procera*, Endophytes, Morphological traits, Mitotic index, Fungal diversity

## Abstract

Endophytic fungi are essential contributors to fungal biodiversity, playing key roles in plant defense against pathogens, alleviation of abiotic stress, and promotion of growth. This study conducted a comprehensive survey of the phenotypic characterization of *Calotropis procera* and its associated endophytic fungi across three regions in Egypt: Qena-Safaga, Qena, and Qena-Kosseir. Positive and significant Pearson correlations among plant morphological traits suggest intrinsic connections. Fungal species diversity exhibited significant variation across the three regions examined. Particularly, the Qena-Kosseir region demonstrated the highest fungal species richness both in soil samples and endophytic fungi. Unique to this region, *Allocanariomyces tritici*, *Aspergillus terreus*, *Chaetomium globosum*, *C. murorum*, *Cladosporium cladosporioides*, *C. sphaerospermum*, *Fusarium proliferatum*, *Penicillium crustosum*, *P. granulatum*, *P. spinuloseum*, and *Roussoella intermedia* were identified as endophytes. Additionally, compared to other regions, the Qena-Kosseir area exhibited the presence of *Aspergillus fumigatus*, *A. ochraceus*, *A. ornatus*, *A. sclerotiocabonarus*, *Drechslera halodes*, *Emericella echinulata*, *Fusarium oxysporum*, and *Macrophomina phaseolina* in soil samples, underscoring its distinct fungal community composition. Furthermore, antimitotic assays using the *Allium cepa* test revealed distinct effects of endophytic extracts on various mitotic stages. Of the 33 treatments, 11 showed an increase in the mitotic index (MI), indicating a potential positive effect on plant growth and cell division. This study offers valuable insights into the diversity and functional roles of endophytic fungi associated with *C. procera*, highlighting their promising applications in sustainable agriculture and plant health management.

**Supplementary Information:**

The online version contains supplementary material available at 10.1007/s00248-025-02503-6.

## Introduction

*Calotropis procera* (Aiton) W. T. Aiton, belonging to the Asclepiadaceae family, is a highly significant medicinal plant that thrives in tropical and subtropical regions worldwide. In Egypt, it is extensively distributed across various phytogeographical zones [1]. *C. procera* demonstrates remarkable adaptability, flourishing in a wide range of soil types, including both acidic and alkaline soils. However, it exhibits a pronounced preference for sandy, well-drained soils. Notably, this plant requires no irrigation, chemical fertilizers, pesticides, or other agronomic inputs, enabling its prolific growth in semiarid and arid environments [2]. It thrives in open areas with minimal grass competition, such as overgrazed pastures and nutrient-deficient soils. Its presence is prominent in disturbed habitats, including roadsides, watercourses, river flats, and coastal dunes [3].

Endophytic fungi are the most vital constituents of a plant’s microbiome. They reside within plants in different associations, and do not exert any detrimental impacts on the plants; they are ubiquitous in nature and can be found residing within the tissues of diverse plant species worldwide [4, 5]. Endophytes participate in mutual interactions with themselves and their host plant, hence modifying the plant’s metabolic capabilities [6].

Ecosystems rely on fungal endophytes because they shield plants from a variety of biotic and abiotic challenges, make them more resilient, and facilitate their adaptation to different environments [7, 8]. One potential ecological function of endophytes is to influence plant growth via antagonistic interactions among fungi. Some fungal endophytes may also play a role in ecosystems by kicking off the biological breakdown of a dying or dead host plant, which begins the recycling of nutrients [9]. Because of their tissue-dwelling nature, endophytes can use the plant nutrients that become available as plants age [10]. Rodriguez et al. [10] introduced the concept of habitat-adapted symbiosis to describe the unique ways in which some fungal endophytes adjust to stress in relation to their specific habitat.

The secondary metabolites produced by endophytic fungi are varied in composition [11]. Among these secondary metabolites are bioactive ones, which the fungus uses as weapons to control pests and diseases and as metabolites for targeted interactions and communication with the plant host. Another advantage imparted by endophytes is the ability to promote plant growth [12]. Furthermore, endophytes are recognized for synthesizing a wide range of secondary metabolites that have important therapeutic applications [13].

The *Allium cepa* test is a widely employed, highly sensitive assay designed to detect chromosomal abnormalities induced by chemical compounds derived from medicinal plants [14, 15]. This assay has a long-standing history in scientific research, having been developed by Levan in 1938 [16]. Initially, it was employed to investigate the effects of various substances on the fidelity of cell division. The *Allium cepa* test remains a robust and effective tool for elucidating the mechanisms by which compounds, whether toxic or nontoxic, either singly or in combination, induce chromosomal alterations [17].

Against this backdrop of intricate plant-endophyte interactions, *Calotropis procera* emerges as a captivating subject for investigation, owing to its medicinal significance and ecological prevalence within tropical and subtropical regions; this plant species holds traditional and contemporary medicinal value, making it an ideal candidate for studying the diversity and functional roles of endophytic fungi within its ecosystem.

The objectives of this study were to investigate the relationship between *C. procera* and its associated endophytic fungi. Specifically, the aim is to explore the potential association between the morphological diversity of *C. procera* plants and the taxonomic diversity of their associated endophytic fungi. Furthermore, comprehensive morphological and molecular characterization of these fungal species was conducted. Additionally, the *Allium cepa* test was also used to assess endophytes’ antimitotic activity.

## Materials and Methods

### Plant Material Collection

**Fig. 1:**
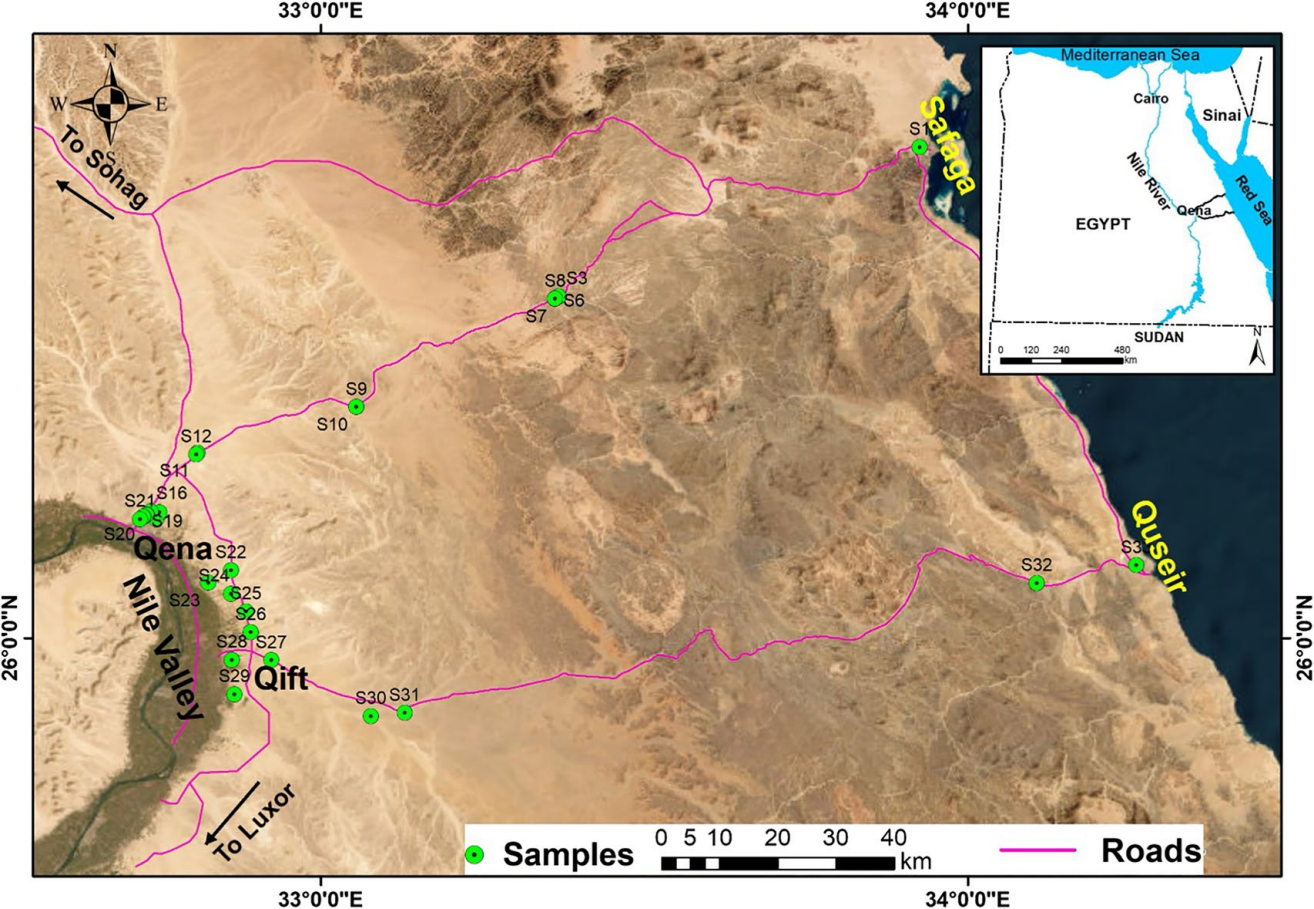
A map of Egypt showing the sites of *Calotropis procera* plants in the Eastern Desert of Egypt

**Fig. 2:**
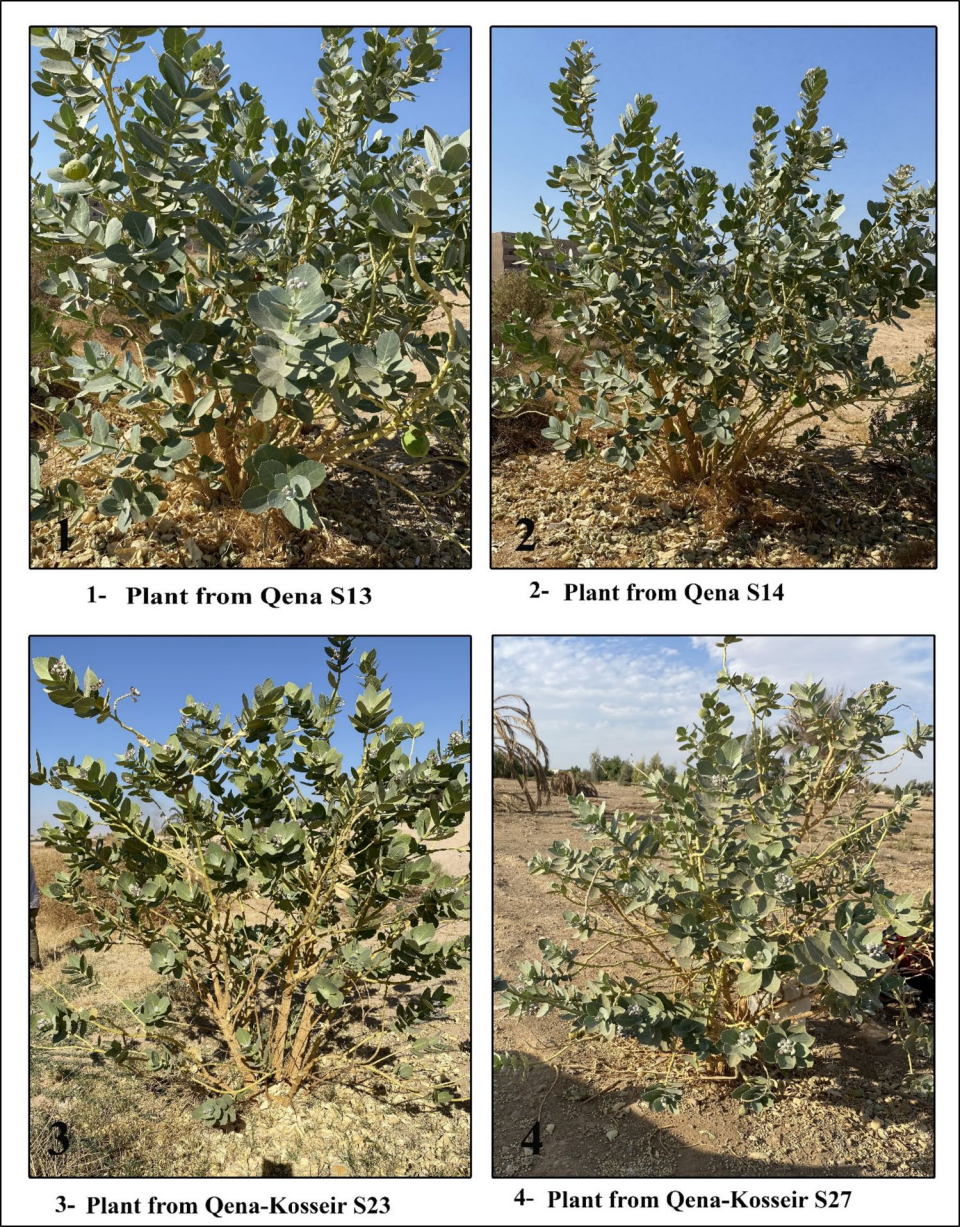
Photograph of some *Calotropis procera* plants collected from some studied sites

Healthy, disease-free *C. procera* samples were systematically collected from natural habitats in the Eastern Desert of Egypt and the Qena governorate during flowering and fruiting, as these stages are likely important for observing morphological traits [18]. All samples were preserved at the South Valley University Herbarium, Botany and Microbiology Department, Qena, and the Aswan University Herbarium (ASW), Egypt, under the voucher number 012000. The geographic coordinates, elevations, and GPS positions of collection sites are shown in Table 1 and depicted on a map of Egypt (Fig. 1). Upon reaching full maturity and blooming stage, morphological traits have been recorded in the plant’s environment, such as the height of the stem, number of flowers per inflorescence, and stem branch number (Fig. 2 and supplementary Fig. 1). A comprehensive examination encompassing nine quantitative morphological traits was conducted to assess the detailed features of a minimum of three plants from each location. The mean value of each quantitative trait, along with its standard error, was calculated. Morphological attributes including leaf fresh weight (FW) (g), leaf dry weight (DW) (g), leaf length (cm), leaf width (cm), leaf index, number of flowers per inflorescence, stem height (cm), and stem branch number were evaluated.

**Table 1:** The sites, GPS location data, and elevation of localities from which *Calotropis procera* were collected

Locations	Region name		GPS Locations data		Elevation (m)
			Latitude	Longitude	
S1	Qena-Safaga	Region No. 1	26° 45′ 32″N	33° 55′ 33″E	40
S2	Qena-Safaga		26° 31′ 41″N	33° 22′ 06″E	501
S3	Qena-Safaga		26° 31′ 41″N	33° 22′ 06″E	500
S4	Qena-Safaga		26° 31′ 29″N	33° 21′ 44″E	499
S5	Qena-Safaga		26° 31′ 29″N	33° 21′ 44″E	498
S6	Qena-Safaga		26° 31′ 29″N	33° 21′ 44″E	500
S7	Qena-Safaga		26° 31′ 29″N	33° 21′ 44″E	501
S8	Qena-Safaga		26° 31′ 29″N	33° 21′ 44″E	492
S9	Qena-Safaga		26° 21′ 27″N	33° 03′ 18″E	228
S10	Qena-Safaga		26° 21′ 27″N	33° 03′ 18″E	227
S11	Qena-Safaga		26° 17′ 12″N	32° 48′ 35″E	148
S12	Qena-Safaga		26° 17′ 03″N	32° 48′ 29″E	146
S13	Qena	Region No. 2	26° 11′ 35″N	32° 44′ 38″E	86
S14	Qena		26° 11′ 02″N	32° 44′ 45″E	86
S15	Qena		26° 11′ 39″N	32° 45′ 03″E	86
S16	Qena		26° 11′ 46″N	32° 44′ 14″E	86
S17	Qena		26° 11′ 27″N	32° 43′ 53″E	83
S18	Qena		26° 11′ 26″N	32° 43′ 52″E	83
S19	Qena		26° 11′ 17″N	32° 43′ 34″E	78
S20	Qena		26° 11′ 16″N	32° 43′ 33″E	78
S21	Qena		26° 11′ 02″N	32° 43′ 15″E	81
S22	Qena		26° 06′ 18″N	32° 51′ 42″E	126
S23	Qena-Kosseir	Region No. 3	26° 05′ 09″N	32° 49′ 36″E	91
S24	Qena-Kosseir		26° 04′ 07″N	32° 51′ 41″E	117
S25	Qena-Kosseir		26° 02′ 29″N	32° 53′ 06″E	109
S26	Qena-Kosseir		26° 00′ 33″N	32° 53′ 31″E	93
S27	Qena-Kosseir		25° 57′ 58″N	32° 55′ 25″E	99
S28	Qena-Kosseir		25° 57′ 59″N	32° 51′ 47″E	95
S29	Qena-Kosseir		25° 54′ 48″N	32° 52′ 00″E	100
S30	Qena-Kosseir		25° 52′ 47″N	33° 04′ 40″E	122
S31	Qena-Kosseir		25° 53′ 05″N	33° 07′ 49″E	132
S32	Qena-Kosseir		26° 05′ 07″N	34° 06′ 26″E	124
S33	Qena-Kosseir		26° 06′ 46″N	34° 15′ 39″E	32

### Fungal Isolation

For the isolation of endophytic fungi, leaves of *C. procera* were sectioned into 5 × 5 mm segments and subjected to surface sterilization protocols [19]. Four disinfected segments were cultured inside each Petri dish containing 20 mL of sterile potato dextrose agar (PDA) supplemented with 0.001 g/L chloramphenicol to thwart bacterial contamination. Incubated at 28 °C for a duration of 1 month, fungal colonies were scrutinized. The plates were examined periodically, and fast-growing fungi were picked.

Thirty-three rhizosphere soil samples were carefully collected near plant roots using sterile spatulas and sealed in pristine polyethylene bags before being expeditiously transported to the laboratory for mycological scrutiny. The dilution plate method [20] was employed for the isolation of fungi from soil samples, and the Petri dishes were incubated at 28 °C for 7 days. The growing colonies were methodically enumerated, purified, and subjected to rigorous taxonomic elucidation. The extended incubation period was chosen to allow for the detection of slow-growing fungal species that might not be visible within shorter incubation times (5–7 days).

### Morphological Characterization of Isolated Fungi

Careful evaluation of the fungal isolates was carried out, including assessment of colony morphology and microscopic examination. Colony characteristics, including coloration, filamentous texture, and mat formation, were documented. Microscopic investigations were conducted on prepared slides of both recently cultured and aged fungal colonies, allowing for the detailed observation of conidial structures, mycelial configurations, and conidiophore morphology, as well as reproductive and non-reproductive structures. Taxonomic classification was conducted following the protocols outlined by Domsch et al. [21], Bensch et al. [22], Hong et al. [23], and Houbraken et al. [24].

### Molecular Identification of Fungi

In instances where morphological characterization proved insufficient, molecular techniques were employed for precise identification. DNA extraction was carried out utilizing the CTAB procedure [25], and DNA quality was assessed using agarose gel electrophoresis [26]. The β-tubulin gene was targeted for amplification using Bt2a and Bt2b primers under the conditions described by Glass and Donaldson [27]. PCR products were visualized via agarose gel electrophoresis stained with ethidium bromide (Supplementary Fig. 2) and subsequently subjected to sequencing at Macrogen, South Korea.

### Diversity of Isolated Fungi from Three Regions

Fungal diversity of endophytic and soil fungi was recorded in each region by using diversity indices: Shannon-Weiner index (*H*') [28], Simpson’s diversity index (*D*) [29], Evenness index (*E*) [30], and Margalef’s index (SR) for species richness [31].

### Preparation of Crude Extracts from Endophytic Fungi

For the preparation of crude fungal extracts, endophytic fungi were cultured in sterile potato dextrose broth (PDB) and incubated at 28 ± 2 °C for 10–15 days. Fungal mycelia were then harvested and separated using Whatman filter paper (15 µm). The resulting filtrate was preserved at -16 °C until further utilization, as per the protocol outlined by Rani et al. [32].

### *Allium cepa* Test for Antimitotic Activity and Cytogenetic Analysis

Fresh onion bulbs procured from the markets of Qena, Egypt, served as the substrate for cytogenetic analysis. Bulbs were induced to sprout by immersion in distilled water until roots measuring 2–3 cm in length emerged. These root tips were then subjected to treatment with endophytic fungal extracts for a duration of 2 h; PDB media served as the control. Subsequently, ten root tips were harvested and preserved overnight in an ethanol-acetic acid mixture (3:1 v/v). Root tip hydrolysis was achieved through treatment with 1 N HCl, followed by the preparation of tinted microscope slides and immersion in Feulgen dye. Mitotic indices, frequency of mitotic stages, and mitotic chromosomal aberrations were quantified following the methodologies delineated by Hassanein et al. [33].

### Data Analysis

Statistical analyses were conducted utilizing the XLSTAT program (Addinsoft, Paris, France) in accordance with the rigorous framework established by Steel and Torrie [34]. One-way ANOVA was employed to scrutinize all morphological measurements, and results were presented as means accompanied by standard error mean (SEM). Treatment means were compared using the LSD test at a significant level of *p* < 0.05. Pearson correlation coefficients were calculated using the SRplot platform [35], as it quantifies the strength and direction of the linear relationship between these traits. PAST 3.22 program [36] was used to construct dendrograms based on Euclidean distance coefficients, employing the Unweighted Pair Group Method with Arithmetic Mean (UPGMA) to analyze the morphological relationships among the plant samples. Principle component analysis (PCA) was conducted on fungal species data utilizing OriginPro, Version 2019b (OriginLab Corporation, Northampton, MA, USA).

## Results

### Plant Morphological Characteristics

**Fig. 3:**
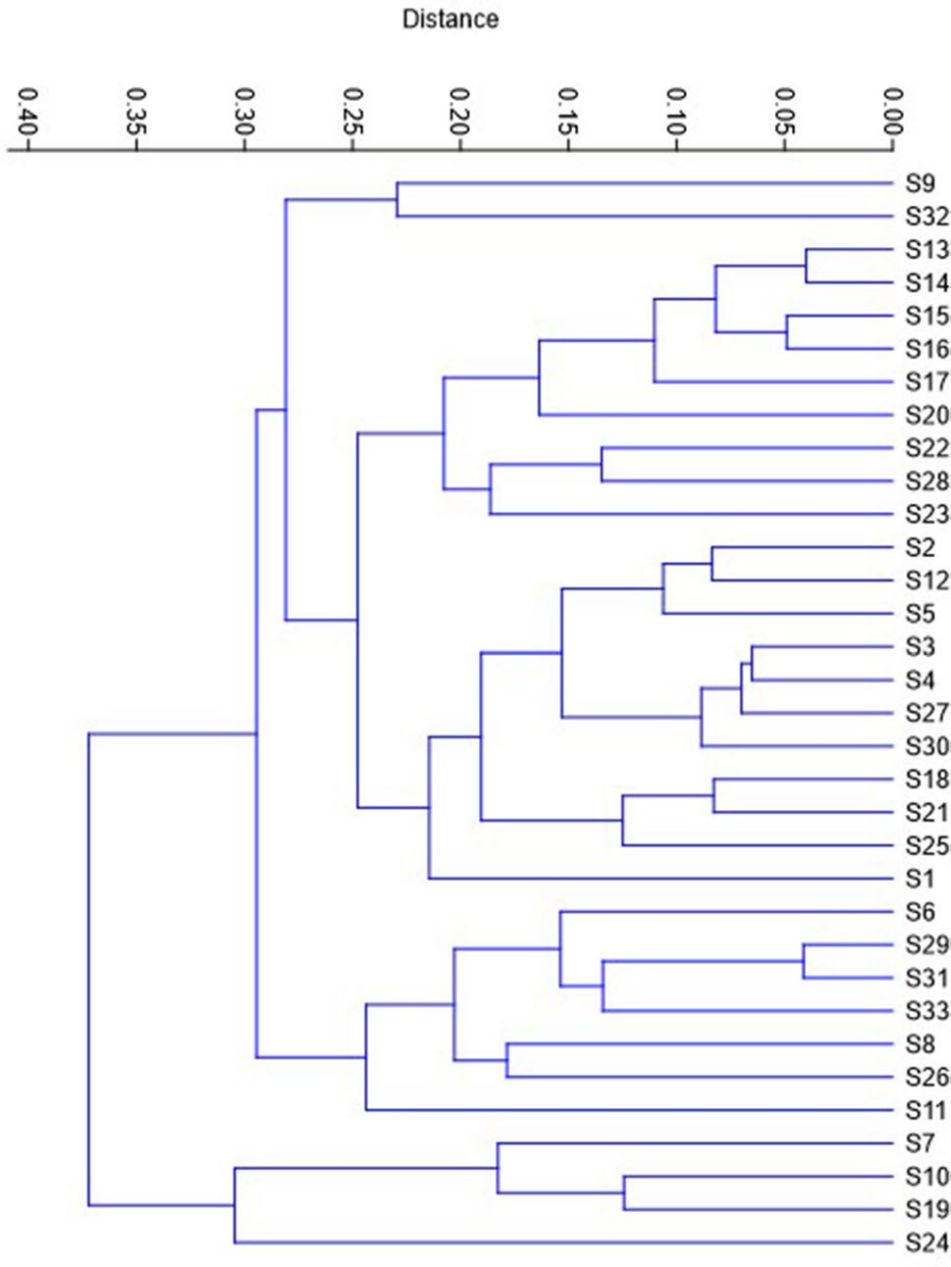
UPGMA distance tree illustrating relationships among *Calotropis procera* plants based on morphological analysis

**Fig. 4:**
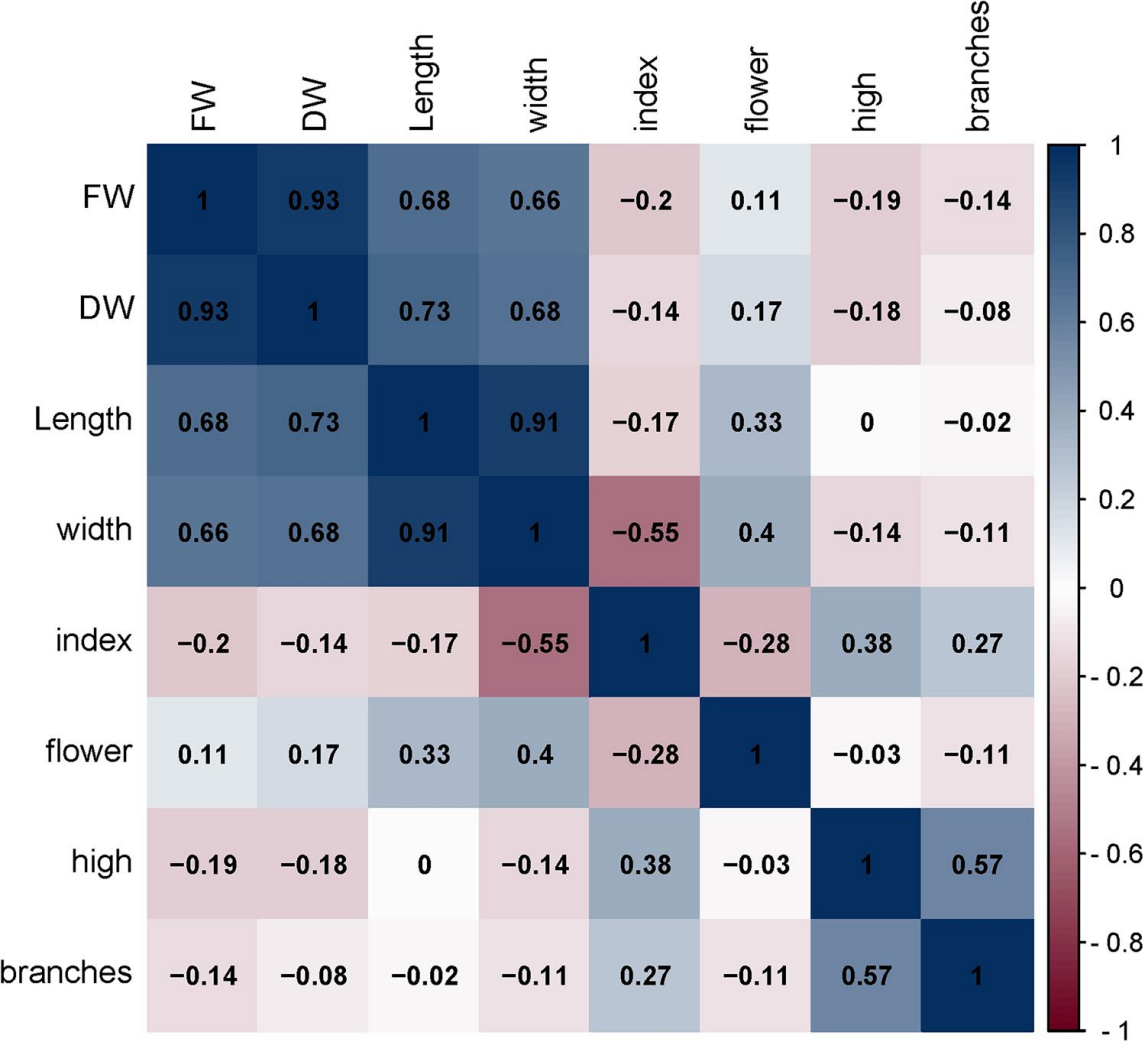
Pearson correlation plot based on morphological traits analysis. Correlation is shown by blue for positive and red for negative. The percentage matrix is shown on the right. In the left-hand plot of the generalized coefficients distribution, each number represents the percentile of the respective correlation coefficients

The morphological characteristics of *C. procera* plants within the study area exhibited considerable variability, as elucidated in Table 2. Notably, the tallest specimens were observed in location S6 (Qena-Safaga), reaching a towering height of 384.3 ± 21.7 cm, followed closely by specimens from S29 and S31 in the Qena-Kosseir region, registering heights of 340.3 ± 18.8 cm and 335.0 ± 21.7 cm, respectively. Conversely, the shortest plant height was documented in S4, measuring 145.3 ± 21.7 cm. Regarding stem branching, specimens from S8 and S33 displayed the highest number of branches (20 ± 1.98), while the lowest number was recorded in S3 and S32 (5 ± 1.98). Flower counts per inflorescence ranged from 11.3 ± 2.12 in S8 to 27 ± 2.12 in S7 along the Qena-Safaga Road. Leaf dimensions varied considerably, with specimens from S24 and S19 in the Qena region exhibiting the highest values of 17.61 ± 0.37 cm and 14.8 ± 0.31 cm for length and 11.1 ± 0.25 cm and 9.98 ± 0.21 cm for width, respectively. Conversely, the lowest values for leaf length and width were observed in S11 along the Qena-Safaga Road, measuring 8.67 ± 0.57 cm and 4.93 ± 0.38 cm, respectively. Leaf indices (leaf length/leaf width) ranged from 1.25 ± 0.06 in S1 along the Qena-Safaga Road to 1.84 ± 0.05 in S23 along the Qena-Kosseir Road. Noteworthy variations were also observed in leaf fresh weight (FW) and dry weight (DW), with specimens from S24 exhibiting the highest FW (9.31 ± 0.38 g) and DW (1.36 ± 0.05 g), while the lowest values were recorded in S32 (FW, 2.33 ± 0.32 g; DW, 0.30 ± 0.05 g). Figure 3 illustrates the results of a cluster analysis based on morphological variations, revealing distinct groupings among the specimens. Notably, specimens from S7, S10, S19, and S24 form one cohesive cluster, while the remaining samples constitute another group. Furthermore, a subcluster within the latter group comprises specimens from S6, S8, S11, S26, S29, S31, and S33. Figure 4 presents a Pearson correlation coefficient plot elucidating the relationships between various morphological traits of *C. procera* plants examined in this study. Positive and significant correlations were observed between leaf FW and leaf DW, leaf length, and leaf width. Additionally, leaf DW exhibited significant positive correlations with leaf length and leaf width. Furthermore, leaf length demonstrated significant positive correlations with leaf width, as well as with stem height, leaf index, and the number of stem branches. Conversely, negative significant correlations were noted between leaf index and leaf width.

**Table 2:** Morphological traits of the *Calotropis procera* plants from various collection sites

Site No	Characters							
	Leaves characters					Flowers No./fluorescence	Stem height (cm)	Stem branches No
	FW (g)	DW (g)	Length (cm)	Width (cm)	Leaf index			
S1	4.53* ± 0.36	0.64 ± 0.05	11.33** ± 0.57	9.10** ± 0.38	1.25** ± 0.06	18.8 ± 1.64	264.3 ± 21.7	12* ± 1.98
S2	4.00** ± 0.32	0.67 ± 0.05	11.40** ± 0.31	7.41** ± 0.21	1.55 ± 0.03	25.4** ± 1.64	253.3* ± 21.7	9 ± 1.98
S3	4.40* ± 0.58	0.65 ± 0.08	10.83* ± 0.57	7.27* ± 0.38	1.50 ± 0.06	19.0 ± 2.12	183.3 ± 21.7	5 ± 1.98
S4	4.37* ± 0.58	0.66 ± 0.08	11.07** ± 0.57	7.13* ± 0.38	1.56 ± 0.06	18.0 ± 2.12	145.3 ± 21.7	7 ± 1.98
S5	4.37** ± 0.32	0.73 ± 0.05	11.66** ± 0.31	7.11* ± 0.21	1.64* ± 0.03	25.3** ± 2.12	251.7* ± 21.7	14** ± 1.98
S6	4.41* ± 0.58	0.69 ± 0.08	11.87** ± 0.57	7.03 ± 0.38	1.69* ± 0.06	15.3 ± 2.12	384.3** ± 21.7	11* ± 1.98
S7	6.15 ± 0.36	0.98** ± 0.05	13.91** ± 0.35	8.94** ± 0.23	1.56 ± 0.04	27.0** ± 2.12	287.3** ± 21.7	16** ± 1.98
S8	2.51** ± 0.32	0.42** ± 0.08	10.25** ± 0.31	5.70 ± 0.21	1.80** ± 0.03	11.3 ± 2.12	301.7** ± 21.7	20** ± 1.98
S9	6.27 ± 0.58	0.69 ± 0.08	8.90 ± 0.57	6.03 ± 0.38	1.48 ± 0.06	15.0 ± 2.12	185.0 ± 21.7	5 ± 1.98
S10	6.80 ± 0.50	1.06** ± 0.07	12.83** ± 0.49	8.98** ± 0.33	1.44 ± 0.05	25.7** ± 2.12	164.3 ± 21.7	10 ± 1.98
S11	6.60 ± 0.58	0.89 ± 0.08	8.67 ± 0.57	4.93* ± 0.38	1.76** ± 0.06	12.0 ± 2.12	313.3** ± 21.7	16** ± 1.98
S12	3.43** ± 0.32	0.68 ± 0.05	11.60** ± 0.31	7.58** ± 0.21	1.54 ± 0.03	20.8* ± 1.64	273.5** ± 13.3	11* ± 1.21
S13	5.70 ± 0.58	0.89 ± 0.08	11.83** ± 0.57	7.07 ± 0.38	1.67* ± 0.06	13.0 ± 2.12	205.0 ± 21.7	13** ± 1.98
S14	6.11 ± 0.58	0.94* ± 0.08	11.80** ± 0.57	6.93 ± 0.38	1.70** ± 0.06	12.7 ± 2.12	176.7 ± 21.7	13** ± 1.98
S15	6.43 ± 0.58	0.98* ± 0.08	10.83* ± 0.57	6.87 ± 0.38	1.58 ± 0.06	12.7 ± 2.12	186.7 ± 21.7	11* ± 1.98
S16	5.94 ± 0.58	1.01** ± 0.08	10.90* ± 0.57	7.03 ± 0.38	1.55 ± 0.06	12.0 ± 2.12	210.0 ± 21.7	12* ± 1.98
S17	5.72 ± 0.32	0.98** ± 0.05	12.89** ± 0.31	7.75** ± 0.21	1.67** ± 0.03	12.0 ± 2.12	260.0* ± 21.7	11* ± 1.98
S18	3.26** ± 0.32	0.46* ± 0.05	10.27* ± 0.33	6.72 ± 0.22	1.53 ± 0.04	12.0 ± 2.12	239.0 ± 21.7	13** ± 1.98
S19	7.41 ± 0.32	1.18** ± 0.05	14.80** ± 0.31	9.98** ± 0.21	1.48 ± 0.03	26.0** ± 2.12	210.0 ± 21.7	12* ± 1.98
S20	6.27 ± 0.41	1.01** ± 0.06	14.77** ± 0.40	9.87** ± 0.27	1.50 ± 0.04	12.0 ± 2.12	210.0 ± 21.7	11* ± 1.98
S21	2.82** ± 0.41	0.35** ± 0.06	9.73 ± 0.40	6.38 ± 0.27	1.53 ± 0.04	12.0 ± 2.12	180.0 ± 21.7	11* ± 1.98
S22	6.80 ± 0.32	0.84 ± 0.05	14.0** ± 0.31	8.73** ± 0.21	1.61 ± 0.03	20.0 ± 2.12	272.7* ± 21.7	9 ± 1.98
S23	5.50 ± 0.50	1.06** ± 0.07	12.43** ± 0.49	6.75 ± 0.33	1.84** ± 0.05	20.0 ± 2.12	227.8 ± 15.4	7 ± 1.40
S24	9.31** ± 0.38	1.36** ± 0.05	17.61** ± 0.37	11.1** ± 0.25	1.59 ± 0.04	18.0 ± 2.12	265.0* ± 21.7	11** ± 1.98
S25	2.86** ± 0.58	0.53 ± 0.08	10.37 ± 0.57	6.73 ± 0.38	1.54 ± 0.06	20.0 ± 2.12	165.0 ± 21.7	12** ± 1.98
S26	2.51** ± 0.41	0.33** ± 0.06	9.33 ± 0.40	5.33 ± 0.27	1.75** ± 0.04	21.7* ± 2.12	293.8** ± 18.8	14* ± 1.71
S27	3.92** ± 0.45	0.56 ± 0.06	11.06** ± 0.44	7.26* ± 0.29	1.53 ± 0.05	19.0 ± 2.12	239.3 ± 18.8	7 ± 1.71
S28	5.85 ± 0.50	0.86 ± 0.07	13.75** ± 0.49	8.35** ± 0.33	1.66* ± 0.05	17.0 ± 2.12	187.3 ± 21.7	6 ± 1.98
S29	3.56** ± 0.32	0.48* ± 0.05	10.78** ± 0.31	6.51 ± 0.21	1.66** ± 0.03	14.0 ± 2.12	340.3** ± 18.8	15** ± 1.71
S30	4.47** ± 0.32	0.58 ± 0.05	11.54** ± 0.31	7.17** ± 0.21	1.61* ± 0.03	20.0 ± 2.12	218.0 ± 21.7	9 ± 1.98
S31	3.33** ± 0.58	0.45* ± 0.08	10.80* ± 0.57	6.33* ± 0.38	1.71** ± 0.06	15.0 ± 2.12	335.0** ± 21.7	16** ± 1.98
S32	2.33** ± 0.32	0.30** ± 0.05	9.06 ± 0.31	5.12* ± 0.21	1.78** ± 0.03	13.7 ± 2.12	220.0 ± 21.7	5 ± 1.98
S33	4.08** ± 0.32	0.51 ± 0.05	12.12** ± 0.31	7.06 ± 0.21	1.72** ± 0.03	18.0 ± 2.12	312.7** ± 21.7	20** ± 1.98

^*^Statistically significant at the 0.05 level, **statistically significant at the 0.01 level. No *, not significant

### Endophytic Fungal Community Associated with *C. procera* Leaves

**Fig. 5:**
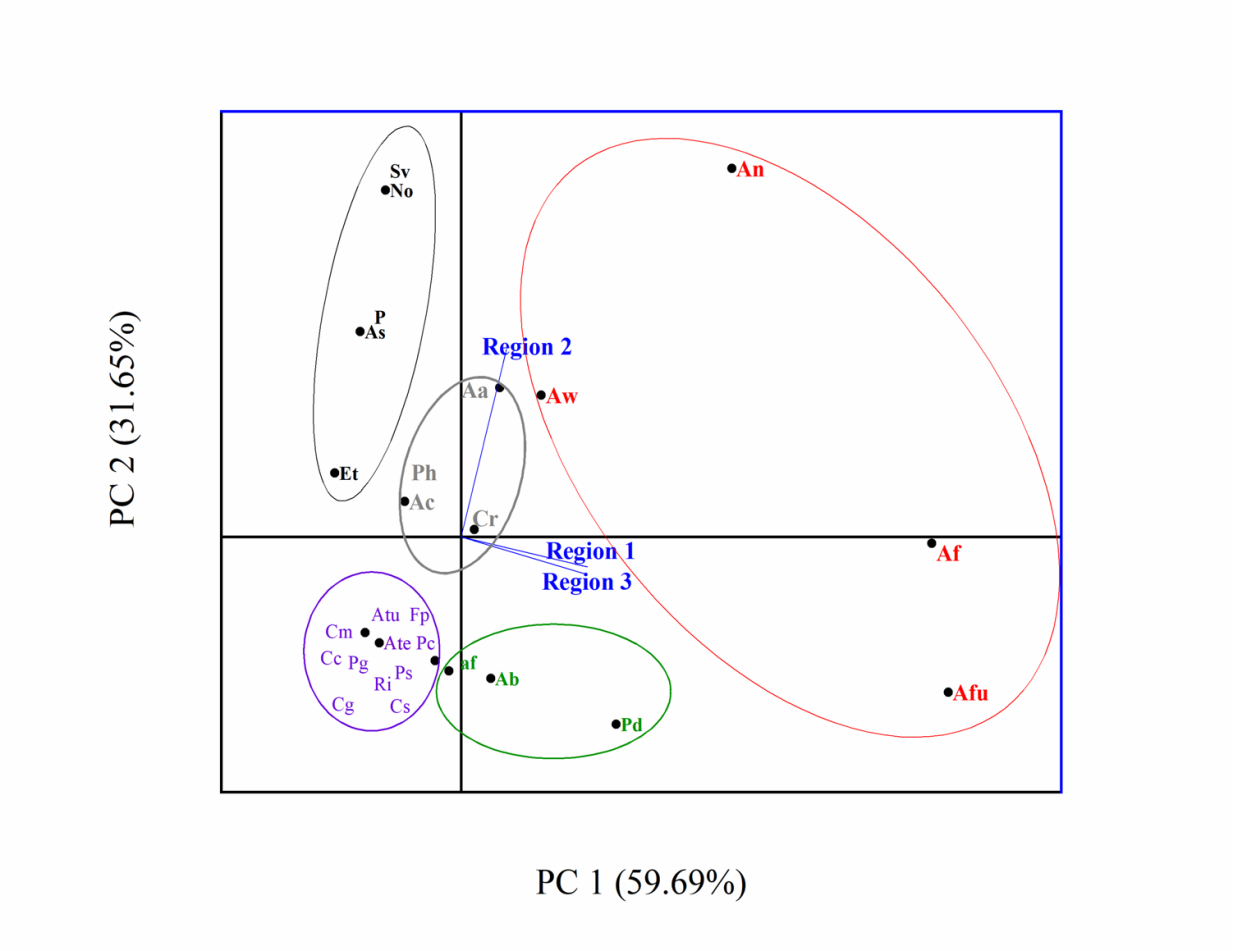
Principal-component analysis (PCA) of number of cases of isolation (NCI) of endophytic mycobiota isolated from *C. procera*. Isolates inside the red circle were found in all three regions; the violet circle comprises isolates found only in region 3; the black circle includes isolates from region 2; the green circle represents isolates found in regions 1 and 3; the gray circle shows isolates found in regions 2 and 3

**Fig. 6:**
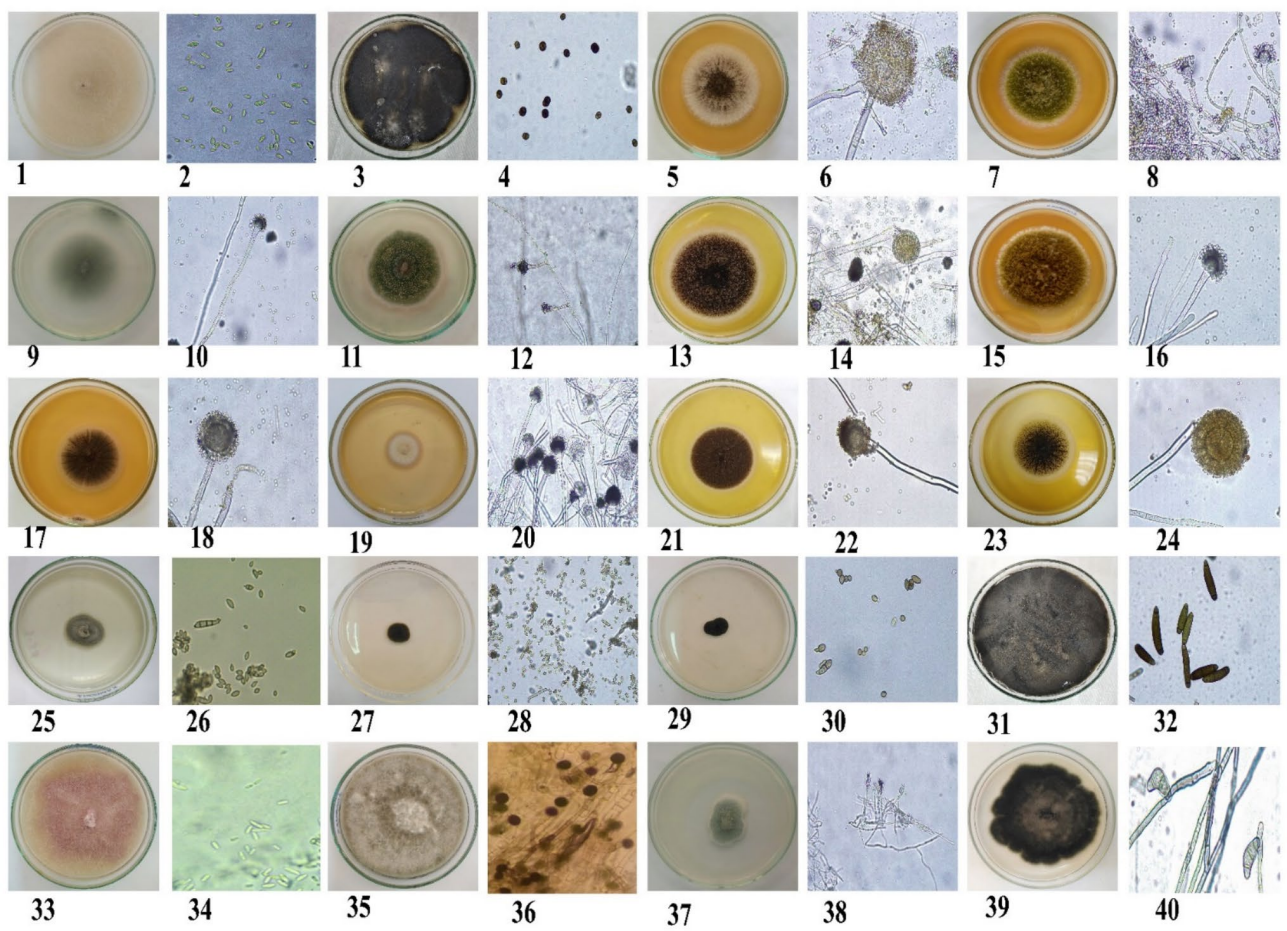
Macro- and microscopic criteria of isolated fungi. 1, 2, *Acremonium sclerotigenum*; 3, 4, *Alternaria atra*; 5, 6, *Aspergillus brasiliensis*; 7, 8, *A*. *flavus*; 9, 10, *A*. *fumigatus*; 11, 12, *Emericella nidulans*; 13, 14, *A. niger*; 15, 16, *A. parasiticus*; 17, 18, *A. sclerotiocabonarus*; 19, 20, *A. terreus*; 21, 22, *A. tubingensis*; 23, 24, *A. welwitschiae*; 25, 26, *Cladosporium cladosporioides*; 27, 28, *C. ramotenellum*; 29, 30, *C. sphaerospermum*; 31, 32, *Drechslera halodes*; 33, 34, *Fusarium proliferatum*; 35, 36, *Nigrospora osmanthi*; 37, 38, *Penicillium duclauxii*; 39, 40, *Paraphaeosphaeria hydei*

Samples were collected from *C. procera* leaves across three distinct regions in Egypt, yielding a total of 33 samples. Morphological analysis revealed the predominant presence of *Aspergillus flavus*, *A. fumigatus*, *A. niger*, and *A. welwitschiae* across regions 1, 2, and 3. Exclusive to region 3 (Qena-Kosseir), *Allocanariomyces tritici*, *Aspergillus terreus*, *Chaetomium globosum*, *C. murorum*, *Cladosporium cladosporioides*, *C. sphaerospermum*, *Fusarium proliferatum*, *Penicillium crustosum*, *P. granulatum*, *P. spinuloseum*, and *Roussoella intermedia* were identified. Region 2 (Qena) yielded isolates including *Acremonium sclerotigenum*, *Exserohilum turcica*, *Nigrospora osmanthi*, *Phoma* sp*.*, and *Stemphylium vesicarium*. *Aspergillus aflatoxiformans*, *A. brasiliensis*, and *Penicillium duclauxii* were found in regions 1 and 3 (Qena-Safaga and Qena regions), while *Alternaria alternata*, *Aspergillus chevalieri*, *Cladosporium ramotenellum*, and *Paraphaeosphaeria hydei* were present in regions 2 and 3 (Table 3 and Figs. 5 and 6). The diversity indices for endophytic fungi on three regions indicated that the value of Shannon-Weiner index (*H*') was 1.91, 2.46, and 2.81 for regions 1, 2, and 3 respectively and region 1 exhibited low diverse when compared by regions 1 and 2. The same result was also ascertained by Simpson’s index which reflects that region 3 had higher diverse (*D* = 0.93) while region 1 was less diverse (*D* = 0.86). Region 3 exhibited higher species richness (RS = 5.39), but on the other side, this region was lower stable or balanced region (*E* = 0.87) than other regions (Table 5).

**Table 3:**
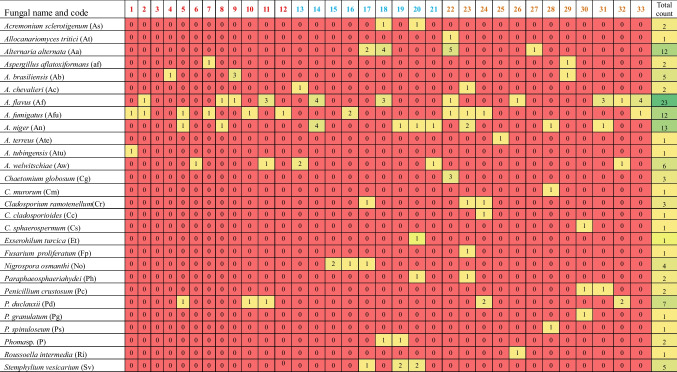
Total counts (calculated per 8 leaf segments for each sample) of various fungal species recovered from leaves *of C. procera*

### Fungal Community Associated with Soil Samples

The mycobiota associated with soil samples revealed a diverse array of fungal species, encompassing 22 species distributed among seven fungal genera. Notably, *Aspergillus tubingensis* and *Emericella nidulans* were ubiquitous, being isolated from soil samples across all three tested regions. Among the recorded species, four were found in two regions: *A. terreus* and *A. versicolor* were identified in regions 1 and 3, *A. flavus* in regions 2 and 3, and *Aspergillus aflatoxiformans* in regions 1 and 2. Conversely, certain fungal species were confined to specific regions; *Aspergillus parasiticus*, *Fusarium nygamai*, *F. semitectum*, and *Penicillium granulatum* were solely isolated from region 1, while *Aspergillus brasiliensis*, *Fusarium solani*, and *Penicillium duclauxi* were exclusively found in region 2. Notably, region 3 boasted the highest species diversity, with a plethora of species including *Aspergillus fumigatus*, *A. ochraceus*, *A. ornatus*, *A. sclerotiocabonarus*, *Drechslera halodes*, *Emericellaechi nulata*, *Fusarium oxysporum*, and *Macrophomina phaseolina*. In general, region 3 showed the highest in all tested biodiversity indices than other regions; it exhibited higher diverse by Shannon-Weiner index value (*H*' = 2.46) and Simpson’s diversity index (*D* = 0.95). At the same time, the higher species richness (RS = 3.94) and higher stability value (*E* = 0.96) were estimated also in region 3 (Tables 4 and 5). Region 3, Qena-Kosseir, exhibited the highest fungal species richness, hosting a diverse array of fungi isolated from both soil samples and endophytic sources. In contrast, region 1, Qena-Safaga, demonstrated the lowest fungal species diversity (Fig. 6 and Table 5).

**Table 4:**
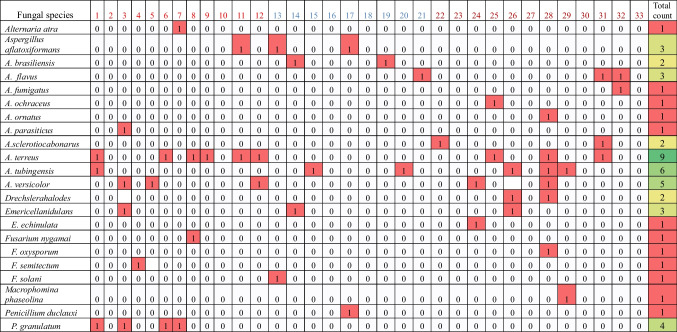
Number of cases of isolation of fungal species recovered from soil samples collected around *C. procera* roots

**Table 5:** Biodiversity indices of the endophytic and soil fungi isolated from *C. procera*, which collected from three regions in Egypt

Fungal community	Location regions	Shannon	Simpson	Evenness	Margalef
Endophyte	Region 1	1.91	0.86	0.91	2.20
	Region 2	2.46	0.91	0.89	3.21
	Region 3	2.81	0.93	0.87	5.39
Soil	Region 1	2.02	0.87	0.87	3.00
	Region 2	1.88	0.93	0.97	2.60
	Region 3	2.46	0.95	0.96	3.94

### Molecular Characterization of Endophytic Fungi

**Fig. 7:**
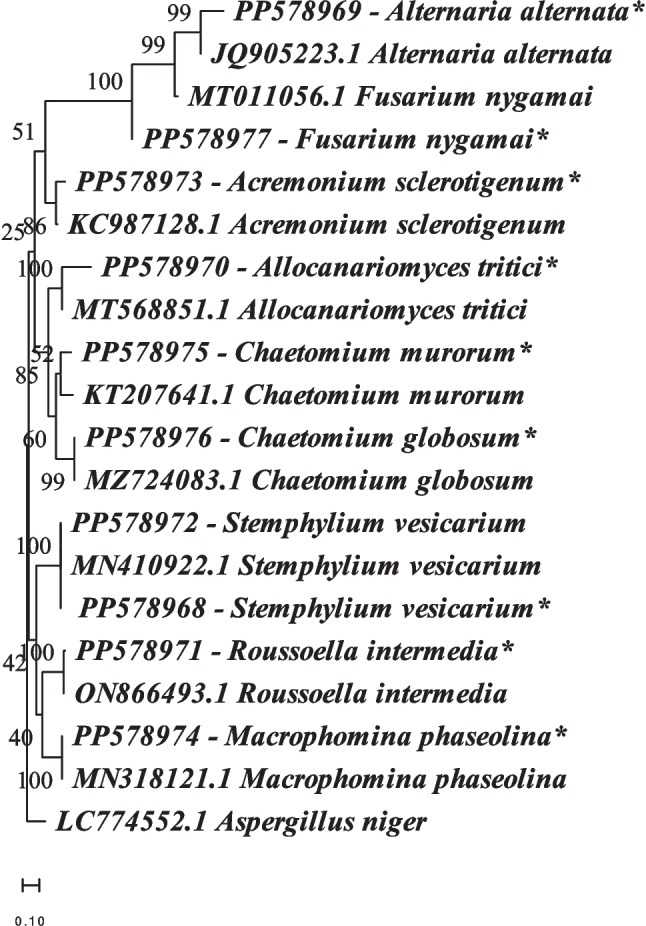
Phylogenetic tree of ten isolates, eight from endophytic fungi and two from the soil, based on the β-tubulin partial gene. It was generated with maximum likelihood and the bootstrap percentage was shown next to the clades, after a run of 1000 replications. With reference sequences obtained from GenBank. The asterisk (*) denotes nodes with high bootstrap support values, suggesting strong phylogenetic relationships between the fungal isolates

Following morphological characterization, ten isolates (eight from endophytic fungi and two from soil) remained unidentified. Consequently, molecular identification was conducted utilizing the β-tubulin partial gene. The obtained sequences were subjected to BLAST analysis using the GenBank database to determine their taxonomic affiliations. Subsequently, Mega X software was employed to construct a phylogenetic tree. The phylogenetic analysis revealed significant matches for several isolates. *Alternaria alternata*, for instance, clustered with an accession number JQ905223.1, exhibiting a bootstrap value of 99%. Similarly, our strain of *Fusarium nygamai* exhibited a 99% bootstrap value when compared with MT011056.1. *Acremonium sclerotigenum* grouped with a strain bearing accession number KC987128.1, with a bootstrap support of 86%. Notably, *Allocanariomyces tritici* displayed a bootstrap value of 100% when compared with MT568851.1. *Chaetomium murorum* clustered with a strain possessing accession number KT207641.1, while *Chaetomium globosum* formed a clade with a strain annotated as MZ724083.1, with both displaying an 85% bootstrap support. *Stemphylium vesicarium*, represented by two isolates, exhibited a 100% bootstrap support when compared with MN410922.1 obtained from GenBank. Similarly, *Roussoella intermedia* formed a clade with an accession number ON866493.1, with a bootstrap support of 100%. Lastly, *Macrophomina phaseolina* displayed a 100% similarity with an accession number MN318121.1. (Fig. 7).

### Genetic Analysis Findings

**Fig. 8:**
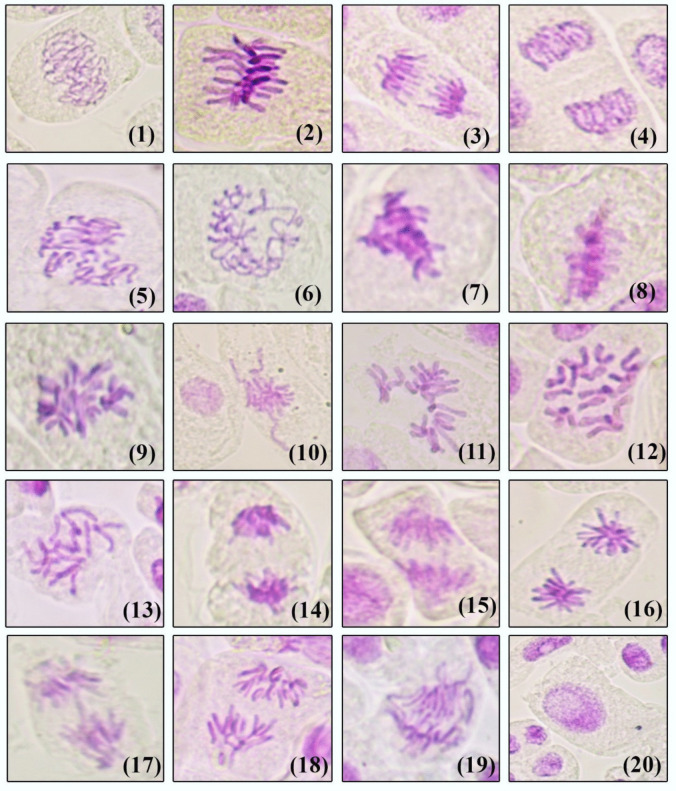
Chromosomal aberrations of *A. cepa* root tips under the treatment of endophyte extraction; (**1**) prophase, (**2**) metaphase, (**3**) anaphase, (**4**) telophase, (**5**, **6**) irregular prophase, (**7**) sticky metaphase, (**8**) diagonal metaphase, (**9**) star metaphase, (**10**, **11**) disturbed metaphase, (**12**, **13**) C-metaphase, (**14**) sticky anaphase, (**15**) diagonal anaphase, (**16**) star anaphase, (**17**) chromosomal bridge in anaphase, (**18**, **19**) disturbed anaphase, and (**20**) polyploidy interphase

Thirty fungal isolates were selected from three regions based on common isolates and dark fungi for investigating their antimitotic activity on *Allium cepa* bulbs. Analysis of the antimitotic activity revealed significant variations among the endophyte extracts (Table 6). Compared to the control, 40% of the treated root tips with endophyte extract exhibited an increase in the mitotic index (MI), while the remaining 60% showed a decrease in the MI. Notably, the extract of *Acremonium sclerotigenum* from S20 demonstrated the highest mitotic index (MI = 6.04%), followed by the extract of *Chaetomium murorum* from S28 (MI = 5.14%). Conversely, some extracts displayed notable inhibition in MI, such as the *Phoma* sp. extract from S19, *Cladosporium sphaerospermum* from S30, and *Allocanariomyces tritici* from S22 (MI = 1.75, 1.78, and 1.8% respectively). Treatment with endophyte extracts influenced the relative frequencies of different mitotic phases (Table 6). Most samples exhibited an increase in metaphase frequencies, with ana-telophase frequencies reaching up to 95.2%, 81.3%, and 66.7% in samples with low MI. The maximum value of total mitotic abnormalities (72.29%) was recorded with *A. flavus* from S14. Interestingly, many endophyte extracts showed no mitotic abnormalities, including *Cladosporium ramotenellum* from S17, *Phoma* sp. and *Stemphylium vesicarium* from S19, *A. niger* from S21, *Allocanariomyces tritici* from S22, *Cladosporium ramotenellum* from S24, and *Chaetomium murorum* from S28. Moreover, no chromosomal abnormalities were observed in the prophase and the percentage of abnormalities in metaphase and ana-telophase did not surpass critical values. Some of the endophyte extracts induced various types of mitotic abnormalities across all mitotic stages (Table 7 and Fig. 8). Common abnormalities in metaphase included C-metaphase, disturbed metaphase, chromosomal stickiness, and diagonal and star metaphase. Ana-telophase abnormalities encompassed disturbed, stickiness, diagonal, star, and chromosomal bridge. Notably, no chromosomal breaks were observed at any stage of abnormalities. All extracts effectively induced chromosomal stickiness, with significant proportions of C-metaphase and disturbed chromosomes observed in *A. flavus* from S14. Additionally, star metaphase and star anaphase were prevalent in *Alternaria alternata* from S22 and *Acremonium sclerotigenum* from S18. Interphase abnormalities such as binucleated and polyploidy interphases were also observed in certain treatments of endophyte extracts, with no micronuclei detected in any treatments. In summary, the study of 30 fungal isolates on *A. cepa* showed varied antimitotic effects, with *Acremonium sclerotigenum* and *Chaetomium murorum* having the highest mitotic index, while *Phoma* sp. and *Cladosporium sphaerospermum* inhibited it. Mitotic abnormalities, such as chromosomal stickiness, were observed, but no chromosomal breaks or micronuclei.

**Table 6:** MI, percentages of mitotic phases and of total abnormal mitotic phases in *A. cepa* root tips under the treatment of endophyte extraction

Site No	Fungal species	MI	% Abnormality	% Prophase		% Metaphase		% Ana-telophase	
				Total	Abn	Total	Abn	Total	Abn
*Control*	Control	3.47	7.58	28.8	0	22.7	1.5	48.5	6.1
*S1*	*A. fumigatus*	2.00	50.34	45.6	0	44.9	44.2	9.8	6.1
*S5*	*A. fumigatus*	3.06	8.82	48.2	1.1	8.8	7.6	42.9	1.1
*S8*	*A. niger*	2.70	31.17	24.7	0	36.4	15.5	38.9	15.6
*S9*	*A. flavus*	2.60	33.33	13.5	0	33.3	13.4	53.2	19.8
*S12*	*A. fumigatus*	3.61	0.78	23.4	0	24.2	0	52.3	0
*S14*	*A. flavus*	5.05	72.29	24.2	0	68.8	67.1	6.9	5.2
*S17*	*Alternaria alternata*	2.78	33.82	51.5	0	36.8	32.4	11.7	1.4
*S17*	*Cladosporium ramotenellum*	2.4	0	0	0	33.3	0	66.7	0
*S17*	*Stemphylium vesicarium*	3.81	13.70	36.5	0	23.7	10.9	39.7	2.7
*S17*	*Nigrospora osmanthi*	5.03	30.48	40.5	2.2	35.3	23.1	24.2	7.4
*S17*	*Alternaria alternata*	2.34	30.91	37.3	0	32.7	20.9	30	10
*S18*	*Phoma*	4.83	13.10	33.1	0	17.2	9.6	49.7	3.4
*S18*	*A. flavus*	3.39	37.98	27.1	0	48.1	37.2	24.8	0.8
*S18*	*Acremonium sclerotigenum*	3.14	37.58	32.1	0	29.1	26.7	38.8	10.9
*S19*	*Phoma sp.*	1.75	0	0	0	4.7	0	95.2	0
*S19*	*Stemphylium vesicarium*	2.87	0	35.4	0	13.9	0	50.6	0
*S20*	*Stemphylium vesicarium*	4.64	22.41	27.1	0	24.7	16.1	48.2	6.3
*S20*	*Paraphaeosphaeria hydei*	2.54	11.65	24.3	0	18.5	8.7	57.2	2.9
*S20*	*Acremonium sclerotigenum*	6.04	11.64	37.9	1.9	10.8	6.3	51.3	4.9
*S21*	*A. niger*	2.47	0	43.2	0	2.7	0	54.1	0
*S22*	*Allocanariomyces tritici*	1.8	0	22.2	0	33.3	0	44.4	0
*S22*	*A. fumigatus*	3.99	7.98	39.5	0.8	5.9	4.6	54.6	3.3
*S22*	*Alternaria alternata*	3.58	44.12	48.5	0	25	23.5	26.5	20.6
*S22*	*Alternaria alternata*	2.96	26.53	66.7	3.7	20.4	14.8	12.9	2.7
*S23*	*Paraphaeosphaeria hydei*	4.68	7.29	23.9	0	28.6	3.5	47.4	4.1
*S24*	*Cladosporium ramotenellum*	2.6	0	0	0	33.3	0	66.7	0
*S26*	*Roussoella intermedia*	3.96	26.88	20.4	0	29.1	22.5	50.5	4.0
*S28*	*Chaetomium murorum*	5.14	0	19.4	0	27.8	0	52.8	2.7
*S30*	*Cladosporium sphaerospermum*	1.78	6.25	12.3	0	6.3	0	81.3	6.3
*S32*	*Penicillium duclauxii*	3.12	19.58	22.5	0	22.9	15.4	54.5	5

**Table 7:** Types and frequencies of abnormal phases after treating of *A. cepa* root tips under the treatment of endophyte extracts

Site No	Fungal species	Interphase abnormalities		Irregular prophase	Metaphase abnormalities					Ana-telophase abnormalities					
		Bi	Poly		C-meta	Dist	Stick	Diagonal	Star	Dist	Stick	Diagonal	Star	Bridge	Free
Control	Control	0.0	0.0	0.0	0.0	1.5	0.0	0.0	0.0	1.5	4.5	0.0	0.0	0.0	0.0
S1	*A. fumigatus*	0.3	0.01	0.0	0.0	4.0	40.3	0.0	0.0	0.7	3.4	0.0	0.0	2.0	0.0
S5	*A. fumigatus*	0.0	0.0	1.2	2.9	2.4	1.8	0.6	0.0	0.0	1.2	0.0	0.0	0.0	0.0
S8	*A. niger*	0.0	0.0	0.0	0.0	6.5	9.1	0.0	0.0	3.9	7.8	0.0	0.0	3.9	0.0
S9	*A. flavus*	0.0	0.0	0.0	0.0	0.0	13.5	0.0	0.0	7.1	7.1	0.0	0.0	5.6	0.0
S12	*A. fumigatus*	0.0	0.06	0.0	0.0	0.0	0.0	0.0	0.0	0.0	0.0	0.8	0.0	0.0	0.0
S14	*A. flavus*	0.0	0.0	0.0	14.3	27.3	23.8	0.4	1.3	0.4	3.0	0.0	0.0	1.7	0.0
S17	*Alternaria alternata*	0.0	0.0	0.0	0.0	0.0	32.4	0.0	0.0	0.0	1.5	0.0	0.0	0.0	0.0
S17	*Cladosporium ramotenellum*	0.0	0.0	0.0	0.0	0.0	0.0	0.0	0.0	0.0	0.0	0.0	0.0	0.0	0.0
S17	*Stemphylium vesicarium*	0.0	0.24	0.0	0.0	0.0	11.0	0.0	0.0	0.0	2.7	0.0	0.0	0.0	0.0
S17	*Nigrospora osmanthi*	0.0	0.02	2.2	0.4	3.0	16.7	2.6	0.4	2.2	1.5	1.5	1.1	0.4	1.8
S17	*Alternaria alternata*	0.0	0.44	0.0	3.6	3.6	13.6	0.0	0.0	3.6	1.8	0.0	1.8	0.0	7.3
S18	*Phoma*	0.0	0.0	0.0	0.7	0.0	9.0	0.0	0.0	0.7	2.1	0.0	0.0	0.7	0.0
S18	*A. flavus*	0.0	0.19	0.0	1.6	31.0	0.8	1.6	2.3	0.8	0.0	0.0	0.0	0.0	0.0
S18	*Acremonium sclerotigenum*	0.02	0.02	0.0	0.6	4.2	21.2	0.6	0.0	0.0	1.8	0.0	9.1	0.0	0.0
S19	*Phoma sp.*	0.0	0.0	0.0	0.0	0.0	0.0	0.0	0.0	0.0	0.0	0.0	0.0	0.0	0.0
S19	*Stemphylium vesicarium*	0.0	0.0	0.0	0.0	0.0	0.0	0.0	0.0	0.0	0.0	0.0	0.0	0.0	0.0
S20	*Stemphylium vesicarium*	0.0	0.0	0.0	0.0	2.9	12.6	0.6	0.0	0.6	5.7	0.0	0.0	0.0	0.0
S20	*Paraphaeosphaeria hydei*	0.0	0.15	0.0	1.0	3.9	3.9	0.0	0.0	0.0	2.9	0.0	0.0	0.0	0.0
S20	*Acremonium sclerotigenum*	0.0	0.0	2.0	0.0	0.0	5.1	1.6	0.0	0.0	3.9	0.6	0.0	0.4	0.0
S21	*A. niger*	0.0	0.0	0.0	0.0	0.0	0.0	0.0	0.0	0.0	0.0	0.0	0.0	0.0	0.0
S22	*Allocanariomyces tritici*	0.0	0.0	0.0	0.0	0.0	0.0	0.0	0.0	0.0	0.0	0.0	0.0	0.0	0.0
S22	*A. fumigatus*	0.1	0.02	0.8	0.4	2.5	0.8	0.8	0.0	0.0	2.1	0.4	0.0	0.8	0.0
S22	*Alternaria alternata*	0.2	0.11	0.0	0.0	2.9	8.8	0.0	11.8	5.9	4.4	1.5	8.8	0.0	0.0
S22	*Alternaria alternata*	0.0	0.0	3.7	0.9	6.5	7.4	0.0	0.9	0.0	1.9	0.0	0.9	0.0	0.0
S23	*Paraphaeosphaeria hydei*	0.0	0.0	0.0	0.0	0.5	2.6	0.0	0.5	0.0	2.6	1.0	0.0	0.0	0.0
S24	*Cladosporium ramotenellum*	0.0	0.0	0.0	0.0	0.0	0.0	0.0	0.0	0.0	0.0	0.0	0.0	0.0	0.0
S26	*Roussoella intermedia*	0.0	0.0	1.1	1.1	1.1	18.3	1.1	1.1	0.0	3.2	1.1	0.0	0.0	0.0
S28	*Chaetomium murorum*	0.0	0.75	0.0	0.0	0.0	0.0	0.0	0.0	0.0	0.0	0.0	0.0	0.0	0.0
S30	*Cladosporium sphaerospermum*	0.0	0.0	0.0	0.0	0.0	0.0	0.0	0.0	0.0	6.3	0.0	0.0	0.0	0.0
S32	*Penicillium duclauxii*	0.0	0.09	0.0	2.9	6.3	5.0	0.4	0.0	0.4	2.5	1.7	0.0	0.4	0.0

## Discussion

This investigation underscores the widespread distribution of *C. procera* across various locales in the Eastern Desert and Qena Governorate of Egypt. Notably, its presence appears more pronounced in urban habitats with considerable human influence compared to arid desert regions [37]. Certain morphological attributes such as large leaf size, tall plant stature, and prolific inflorescence are closely linked to its distribution patterns. These traits are instrumental in facilitating the plant’s dispersion. Al-Sodany et al. [37] posit that *C. procera* may readily naturalize across diverse habitats within their study area, potentially influencing the local vegetation. The plant’s abundance of minute seeds, capable of wind dispersal and rapid germination, coupled with its robust competitive capabilities and substantial adult dimensions, likely contribute to its extensive distribution. Phenological studies suggest that *C. procera* has proliferated globally for both ornamental and commercial purposes.

Its functional attributes, including large leaf structures, wind-dispersed seeds, bisexual flowers, and pollinator-attracting capabilities, further augment its invasive potential [38]. Notably, *C. procera* demonstrates adaptability and thriving tendencies in the urban environs of South Cairo, Egypt, owing to its functional and phenological traits [39]. It was noticed that the plant samples that were collected from the Qena-Kosseir region were characterized by fresh and succulent leaves as compared with other regions. This is an indicator that the water content in this region helped the growth of plants and subsequently the development of endophytic and soil fungi. A unique strategy for reducing the impacts of drought stress in colonized plants can be found in the relationship between plant survival in desert environments and microbial interactions. Fresh and dry biomass, leaf area, root/shoot ratio, relative water content, and membrane stability index were all enhanced by the symbiotic connection between fungal endophytes and maize roots, as reported by Bakhshi et al. [40].

Regarding endofungal species associated with *C. procera* leaves, *Aspergillus flavus*, *A. fumigatus*, *A. niger*, and *A. welwitschiae* emerged as predominant species. Additionally, a total of eight fungal species, including *Aspergillus flavus*, *A. niger*, *Aspergillus* sp., *Penicillium sublateritium*, *Phoma chrysanthemicola*, *P. hedericola*, *Phoma* sp., and *Candida albicans*, were reported in *C. procera* in India [41]. Furthermore, *Aspergillus terreus*, isolated in this study, aligns with findings by Sukar and Elazab [42], highlighting the rich endophytic diversity of *C. procera* compared to other medicinal plants collected in Egypt. The prevalence of fungal species such as *A. niger* and *Beauveria bassiana*, followed by *A. terreus* and *Trichoderma harzianum*, corroborates with prior research [43], which emphasized the significance of endophytes fungi in *C. procera* ecosystems, after testing the antifungal activity of isolated endophytic fungi against four phytopathogens. *Chaetomium globosum* and *Myrothecium verrucaria* exhibited inhibition rates of 20 to 80%.

The diverse array of endophytic fungi associated with *C. procera* likely plays a pivotal role in modulating its phenotypic traits, including its prominent leaf size, robust stature, and abundant flowering capacity. These fungi may produce bioactive secondary metabolites that enhance the plant’s growth and competitive advantages, particularly in challenging environments like protection from microbial or insect diseases. Additionally, certain fungal species are known to improve nutrient uptake and bolster stress resilience, which may account for *C. procera*’s ability to thrive across both urbanized areas and arid landscapes [44]. This symbiotic interaction suggests that the endophytic fungal community significantly influences the plant’s adaptive strategies, contributing to its rapid spread and invasive potential.

Environmental variables, including weather and local flora, are influencing the variety and organization of fungal endophytes [45]. In the rhizosphere soil of *C. procera*, a total of 22 fungal species were identified. *Aspergillus tubingensis* and *Emericella nidulans* were detected across all three regions, consistent with previous findings in Chinese and Egyptian soils [46, 47]. Additionally, *Aspergillus aflatoxiformans*, *A. flavus*, *A. terreus*, and *A. versicolor* were isolated from two regions, aligning with the predominance of *A. flavus* and *A. terreus* in various soil types worldwide [48–50]. Notably, the remaining species were previously isolated from diverse soils, highlighting the ecological adaptability of these fungal species. Potential explanations for this trend include the distinct environmental conditions of the region, such as soil composition, climate, vegetation, and elevation which may create a more conducive environment for fungal growth. Additionally, microhabitat variations within the region could further support higher fungal colonization, and region-specific fungi enhance the understanding of plant growth and fungal biogeography. Our findings are consistent with previous studies highlighting the significant role of environmental factors, including soil composition, moisture levels, and local flora in shaping endophytic fungal diversity [51].

Microbial endophytes may help plants grow well in bad soil by producing phytohormones and breaking down dangerous chemicals. Endophytes help plants deal with a lot of things, like salt, heat, dryness, heavy metal stress, and nutritional deficiencies. To make agriculture and farming more sustainable and less harmful to the environment, study needs to keep focusing on microbial endophytes [8] and find ways to make plants and crops more productive.

Our findings reveal heightened mitotic activity in the root tips of 11 out of 30 treatments. Although the decrease in mitotic index compared to the control group was not significant, most treatments did not exhibit mitotic aberrations. While previous studies have indicated that endofungal extracts with antimitotic properties reduce the mitotic index, our results demonstrate a contrasting effect [52–54]. Chromosomal abnormalities observed in our study have been documented in previous research following exposure to endophytic fungal extracts, indicating the potential cytotoxic effects of these compounds [53, 54]. Considering *C. procera*’s adaptation to harsh environmental conditions, these chromosomal alterations may represent an adaptive response to enhance tolerance against abiotic stresses common in some environments [55]. The secondary metabolites of certain fungal species may account for the variations in the mitotic index observed, underscoring the multifaceted roles of fungal endophytes in plant growth and development. Four antifungal metabolites, griseofulvin, dechlorogriseofulvin, 8-dihydroramulosin, and mellein, were isolated from *Nigrospora* sp., with the latter three reported for the first time. Griseofulvin showed strong in vitro activity against eight plant pathogenic fungi [56]. Prajapati et al. [57] suggest that endophytic fungi enhance plant growth through phytohormone production, nutrient solubilization, and stress tolerance, while also acting as biocontrol agents. Chromosome stickiness, a common observation in our study, may result from physical adhesion related to the protein-based matrix of chromatin material during chromosomal condensation. This phenomenon, along with the occurrence of star metaphase, reflects mitotic disturbances that could affect cell division and genetic stability. These disruptions suggest potential cytotoxicity and alter the biological activity of the endophyte extracts. Various spindle-related abnormalities, including C-metaphase, disturbed metaphase, and star-shaped phases, were also noted, alongside anaphase bridges possibly formed due to chromosomal stickiness during anaphase. Notably, no clastogenic or mutagenic effects were observed, as evidenced by the absence of chromosomal breaks or micronuclei formation.

This study offers valuable insights into the diversity and functional roles of endophytic fungi associated with *Calotropis procera*. The widespread distribution and adaptability of *C. procera* are attributed to its distinctive phenotypic traits and beneficial associations with diverse fungal endophytes, which enhance the plant’s resilience to environmental stresses and support growth and competitive advantages. Cytogenetic analyses further reveal the influence of fungal metabolites on plant development. These findings underscore the crucial role of microbial interactions in shaping the success of *C. procera*, with promising applications in sustainable agriculture and plant health management. Additionally, understanding the role of microbial interactions in *Calotropis procera* provides insights into managing invasive species and their ecological impact. Next-generation sequencing (NGS) technologies should be used in the future to study endophytic fungi for taxonomic identification to record the genetic variations between fungal species and various ecological aspects, including interactions with hosts, lifestyle and functional heterogeneity, evolution, biosynthetic pathways of secondary metabolites, resistance to heavy metals and high salinity, and communication with other fungi and bacteria [58]. Further research should explore the potential of specific bioactive compounds produced by fungi, as these could have significant pharmacological applications.

## Supplementary Information

Below is the link to the electronic supplementary material.

Supplementary file1 (DOCX 1681 KB)

## Data Availability

No datasets were generated or analysed during the current study.
