# Supplementary material for: Diversity and Symbiotic Associations of Endophytic Fungi in Calotropis procera (Aiton) W.T. Aiton (Asclepiadaceae) Across Three Egyptian Regions: Phenotypic Characterization and Mitotic Activity
Source: Microb Ecol. 2025 Mar 5;88:10. doi: 10.1007/s00248-025-02503-6 (PMC11882703; doi:10.1007/s00248-025-02503-6)
Supplement: Supplementary file 1 [file 248_2025_2503_MOESM1_ESM.docx]

**Supplementary figure 1:** Photograph of some *Calotropis procera* plants collected from the studied sites. (1-4) Region No. 1 from Qena-Safaga, (5-8) Region No. 2 from Qena, (9-12) Region No. 3 from Qena- Kosseir, and some morphological traits (13-14) the Flowers No./ Fluorescence in Qena-Safaga, (15-16) Stem branches number in Qena- Kosseir region.

**Supplementary figure 2: 1-6:** Polymerase chain reaction products achieved from endophytic fungi species isolates by using primer pair Bt2a/Bt2b. M: 100 bp molecular size marker, lanes from 1 to 6, PCR products of *Acremonium sclerotigenum*, *Alternaria alternata*, *Allocanariomyces tritici*, *Chaetomium globosum*, *Stemphylium vesicarium* and *Roussoella intermedia* respectively.
